# Sero‐Prevalence and Molecular Detection of *Mycobacterium bovis* in Cattle at Sylhet Division of Bangladesh

**DOI:** 10.1002/vms3.70531

**Published:** 2025-07-28

**Authors:** Md. Atik Faysal, Md. Shahidur Rahman Chowdhury, Fatema Yeasmin Tanni, Hemayet Hossain, Khadiza Akter Brishty, Md. Bashir Uddin, Md. Masudur Rahman, Md. Mahfujur Rahman, Md. Mukter Hossain

**Affiliations:** ^1^ Department of Medicine, Faculty of Veterinary Animal and Biomedical Sciences, Sylhet Agricultural University Sylhet Bangladesh; ^2^ School of Veterinary Medicine and Biomedical Sciences, University of Nebraska‐Lincoln Lincoln Nebraska USA; ^3^ Department of Biochemistry University of Nebraska‐Lincoln Lincoln Nebraska USA; ^4^ Department of Anatomy and Histology, Faculty of Veterinary Animal and Biomedical Sciences, Sylhet Agricultural University Sylhet Bangladesh; ^5^ Department of Zoology (GSSC) University of Dhaka Dhaka Bangladesh; ^6^ Department of Pathology, Faculty of Veterinary Animal and Biomedical Sciences, Sylhet Agricultural University Sylhet Bangladesh

**Keywords:** antibodies | bovine tuberculosis | molecular detection | public health | serum ELISA

## Abstract

**Background:**

*Mycobacterium bovis*, the cause of bovine tuberculosis (bTB), is a contagious, notifiable, chronic bacterial disease that causes economic losses. bTB remains a significant zoonotic threat, particularly in resource‐limited settings where reliable prevalence data are still lacking.

**Objectives:**

This study aimed to determine the serological prevalence and molecular detection of bTB from different commercial and individual dairy farms of Sylhet and Sunamganj districts in Bangladesh.

**Methods:**

A cross‐sectional study was conducted on a total of 250 blood and 250 milk samples, which were collected across selected dairy farms. Serological testing was performed using indirect ELISA, and molecular detection of *M. bovis* was carried out through PCR. Associations between infection status and potential risk factors were evaluated using chi‐square tests and logistic regression models.

**Results:**

The overall prevalence of *M. bovis* was 10.6%. Molecular detection through PCR revealed a higher prevalence in milk (14.8%) compared to blood (6.4%) samples. Serological testing showed that the prevalence in Sylhet district was lower (5.3%) than in Sunamganj district (12.0%). The highest prevalence was observed in diarrhoeic animals (19.49%), followed by female cattle (13.58%) and Holstein Friesian cows (14.0%). Cattle aged 1–3 years showed a notable prevalence of 17.51%.

**Conclusions:**

The presence of *M. bovis* in dairy cattle herds was confirmed by both molecular and serological methods, highlighting the endemic nature of bTB in the study area. These findings underscore the importance of routine surveillance, combining diagnostic approaches and implementing effective control measures to reduce the burden of bTB in dairy production systems.

## Graphical abstract

Five hundred samples (250 milk and 250 blood samples) were collected by using a simple random sampling method. Following collection, blood was let to clot by being left undisturbed at room temperature for 15–30 min, and serum was separated by centrifugation. Extraction of DNA from milk and blood was performed accordingly. PCR was conducted using the target primer sequences and identified by electrophoresis by visualizing the *M. bovis*–specific band at 150 base pairs.

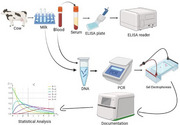

## 1 Introduction

Bovine tuberculosis (bTB), caused by *Mycobacterium bovis*, is a significant zoonotic disease affecting cattle and humans, particularly in low‐ and middle‐income countries. The disease poses a threat to public health and economic productivity due to its impact on livestock and potential transmission to humans through infected milk (Khairullah et al. 2024; Tadesse 2017). In humans, bTB can cause extrapulmonary tuberculosis, and the consumption of unpasteurized milk is a common route of transmission, especially in regions where pasteurization is not routinely practised (Quadri et al. 2021; Dubey et al. 2020).

Bovidae, both domesticated and feral, have been found to carry the species. Goats, sheep, pigs, horses, cats and dogs, as well as fennec foxes, bison, buffalo, badgers, wild and feral pigs, antelope, camels, humans and non‐human primates have all been diagnosed with the disease (Sa'idu et al. 2015). Cattle migrations, especially those coming from areas having a history of bTB, are the most reliable indicator of disease outbreaks (Gilbert et al. 2005). Cattle having bTB first show no symptoms, so farmers often don't suspect an outbreak until they get their herds tested. Crossbred, Holstein Friesian and indigenous cows are the most prominent breeds in Bangladesh's dairy sector. The majority of dairy farming in the region involves rearing these three types of cattle, as highlighted in several studies (Al Emon et al. 2024; Farabi et al. 2024). One of the most significant zoonotic diseases affecting both dairy cattle and humans in this region is bTB (Hossain et al. 2024). bTB is primarily transmitted through inhalation of aerosols from infected animals and ingestion of contaminated dairy products. Infected cattle can shed *M. bovis* in respiratory secretions, milk and other bodily fluids, facilitating transmission to other animals and humans (Khairullah et al. 2024; Tadesse 2017).

*M. bovis* (bovine tubercle bacillus), the aetiological agent of bTB, is a member of the *Mycobacterium tuberculosis* complex (Sa'idu et al. 2017). bTB can occur anywhere, but it is most prevalent in low‐income countries where surveillance and control measures are insufficient (Gumi et al. 2011). Cattle are particularly susceptible to infection with *M. bovis* during their first year of life, often experiencing a prolonged subclinical period with little or interrupted shedding. This is eventually followed by advanced disease, which presents symptoms in a small percentage of affected cattle (Brooks‐Pollock et al. 2014). Although the bovine tubercle bacillus is the root of bTB in cattle, it is, however, common to refer to bovine tubercle bacillus strains regardless of the host. In humans, tuberculosis (TB) is individualized by persistent respiratory distress, overall physical imbalance, depleting and nocturnal shivering with a minor temperature (Thoen et al. 2009). A systematic review and meta‐analysis reported a wide range of *M. bovis* prevalence in human TB cases, from 0.4% to 76.7%, with an overall pooled prevalence of 12.1% from various studies (Taye et al. 2021). In developing countries, *M. bovis* accounts for 10%–15% of new human TB cases, whereas in developed countries, it is responsible for 1%–2% of cases (Dubey et al. 2020).

The prevalence of bTB varies significantly depending on the diagnostic methods, animal populations and geographic regions. A study in the Gangetic delta region of West Bengal, India, used both tuberculin tests and PCR, finding a higher prevalence in exotic crossbred animals (34.6%) compared to indigenous cattle (10.5%) (Das et al. 2018). In Bangladesh, the tuberculin skin test (TST) has been utilized to assess the prevalence of bTB. In the Pabna area, the TST positivity rate for cattle was 5.9%, whereas in the Mymensingh district, it was 3.05%. The prevalence of bTB in breeding bulls was estimated at 27.5% at the Central Cattle Breeding and Dairy Farm and 7.1% at the Bangladesh Livestock Research Institute farm in Savar, Dhaka (Biswas et al. 2017; Islam et al. 2021). Several serological studies estimated the sero‐prevalence of bTB at 7.5% in dairy cattle in Chattogram, 5.9% in Mymensingh and 7.8% in Sirajganj, highlighting the importance of serological tools in understanding disease prevalence and identifying risk factors (Hossain et al. 2023; Mondal et al. 2014; Mahmud et al. 2014).

The above studies have reported varying prevalence rates depending on the diagnostic approach, animal population and geographic region. This variability underscores the importance of using both molecular and serological tools to obtain a comprehensive understanding of bTB prevalence and associated risk factors. However, data on bTB prevalence using both PCR and ELISA in dairy cattle in Sylhet, Bangladesh are scarce. Therefore, this study aims to estimate the prevalence of bTB using molecular and serological tools and to identify associated risk factors, which is crucial for designing awareness campaigns and bTB control programs.

## 2 Materials and Methods

### 2.1 Study Area and Study Design

**FIGURE 1:**
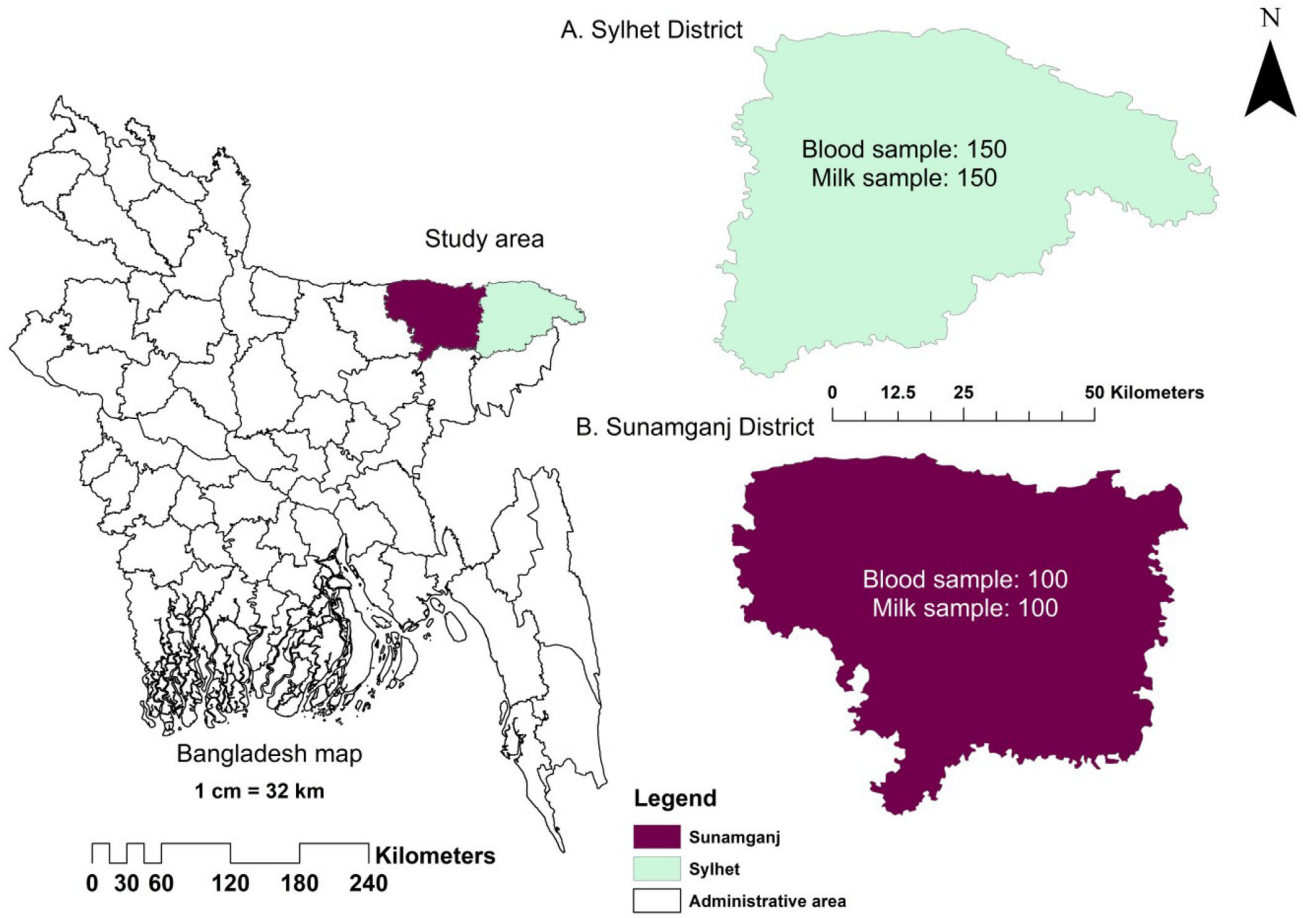
The map showing the study area with specific sample numbers. (A) Sylhet district showing specific sample number; (B) Sunamganj district map showing specific sample number of this study. *Source*: The map was generated using ArcMap 10.8 software.

This study was carried out in the Sylhet Division, the northeastern region of Bangladesh (Figure 1). Two out of the four districts, Sylhet and Sunamganj, were purposefully selected for this cross‐sectional study due to their comparatively higher cattle densities and milk productivity (source: Upazila Livestock Office, Sylhet and Sunamganj). The study included both semi‐intensive and extensive dairy farms with Holstein Friesian, crossbred and indigenous cows, considering factors such as age, sex and health conditions. The study was conducted from January 2021 to December 2022.

### 2.2 Sample Collection

A total of 500 samples—comprising 250 milk and 250 blood samples—were collected using a simple random sampling technique. Initially, extensively managed and semi‐intensive cattle farms located in the Sylhet and Sunamganj districts were identified and listed. Farms were considered eligible for inclusion if they met the following criteria: (i) a minimum herd size of 10 cows, (ii) operational for at least 3 years and (iii) owner consent for milk and blood sample collection. After confirming eligibility, individual cattle within each farm were assigned unique identification numbers, and the required number of animals was selected using a random number generator (RNG) to ensure unbiased sample selection.

Milk was aseptically collected into sterile 15 mL Falcon tubes with a volume of 10–15 mL and stored at 4°C until analysis. Blood was collected aseptically directly from jugular vein in plain vacutainer tubes and stored at −20°C.

Sample size was calculated on the basis of the following formula and reference prevalence (Islam et al. 2021):

$$n=\frac{1.96^{2}P\left(1-P\right)}{d^{2}}$$

where *p* (expected prevalence) = 0.10, and *d* (margin of error) = 0.05 at 95% confidence interval (CI). On the basis of these assumptions, a minimum of 139 samples is required to conduct this study. However, this study was conducted with a total of 500 samples.

### 2.3 Serum Separation From Blood Samples

Following the collection of whole blood, it was allowed to clot by being left undisturbed at room temperature for 15–30 min. The clot was removed by centrifugation in a refrigerated centrifuge at speeds ranging from 1000 to 2000 × *g* for 10 min. The resulting supernatant was designated as serum. After centrifugation, the clear supernatant was transferred into a polypropylene tube. The serum was then stored at −20°C for further analysis.

### 2.4 Serological Examination of *M. bovis*

Serum samples were tested for antibodies against *M. bovis* using a commercial ELISA kit (Cat. No. SL0127Bo, Sunlong Biotech Co. Ltd., Hangzhou, Zhejiang, China). The absorbance was measured at 450 nm using a microplate reader. Samples were considered positive if the optical density (OD) value exceeded the threshold as defined by the manufacturer. The results were interpreted using the cutoff values provided by the manufacturer: S/P < 0.25—considered negative for bTB; S/P ≥ 0.268—considered positive for bTB. Values (S/P) between 0.25 and 0.268 were classified as inconclusive, necessitating further testing for confirmation.

### 2.5 Extraction of Bacterial Genomic DNA

*AddPrep Genomic DNA Extraction Kit* (ADD BIO INC, Daejeon, Republic of Korea, Cat. No. 10023) was used for serum samples, following the manufacturer's instructions. Similarly, DNA was extracted from approximately 10 mL of milk samples. This volume was centrifuged to concentrate the bacteria, and then the pellet was used for DNA extraction. Briefly, 20 µL of proteinase K and 200 µL of lysis buffer were added to 200 µL of the sample solution and incubated at 56°C for 10 min. Following the incubation step, a total volume of 200 L of absolute ethanol was added to the lysate. After that, the sample was washed and centrifuged in accordance with the manufacturer's instructions. The elution buffer provided in kit was used (100 µL) to elute the nucleic acid.

### 2.6 PCR Amplification

**FIGURE 2:**
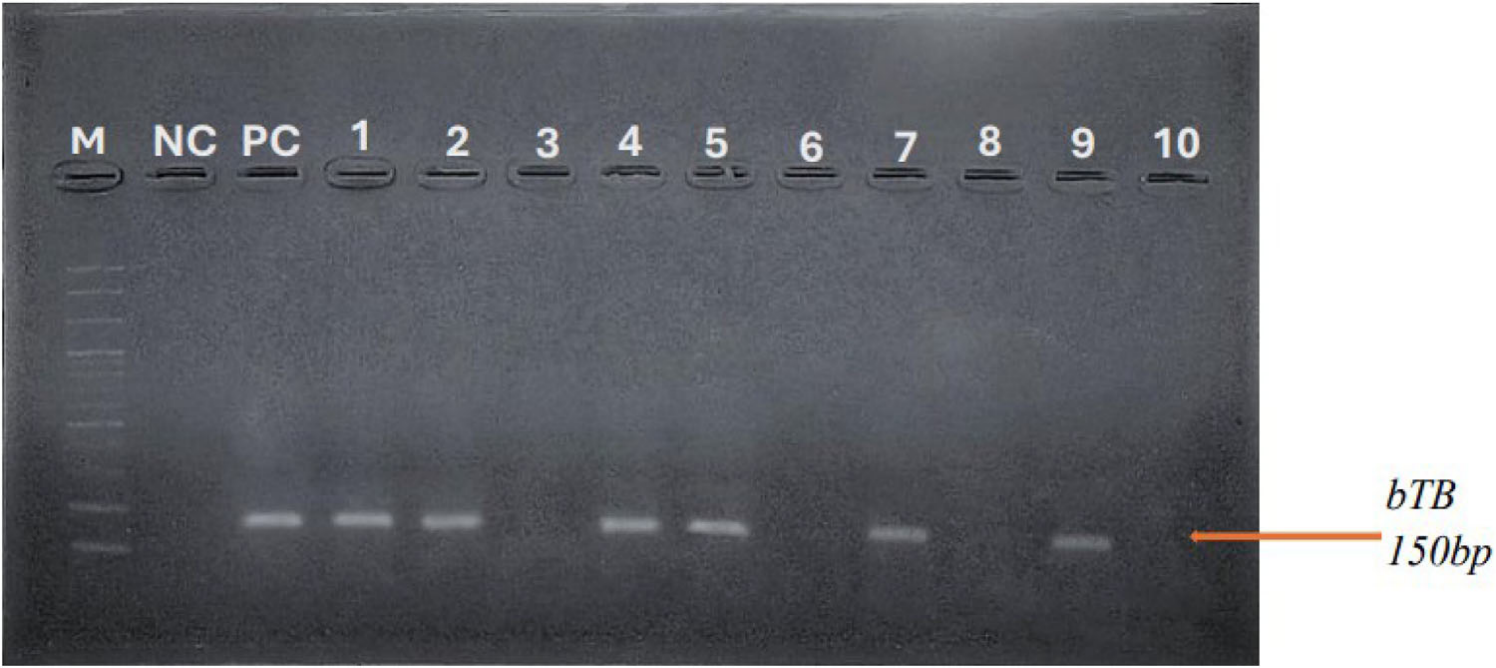
PCR amplification of *M. bovis* (150 bp). M, Marker 100 bp DNA ladder. bTB, bovine tuberculosis; NC, negative control; PC, positive control.

PCR was conducted using the following IS6110 primer pair: (5′ GGGGATCTCAGTACACATCGATGTTCAGCGAG 3′) and (5′ TGCCGGGTTTGATCAIIICGGTCTTGTA 3′). The target sequence (150 bp) was amplified by PCR using certain primer pairs on the isolated bacterial DNA. Primers were utilized in a final reaction volume of 20 µL using 10 µL of master mix (2× conc. with UDG). Each reaction contained 5 µL of extracted DNA, 10 µL of master mix and 5 µL of primers (forward and reverse). This mixture was mixed very well by repeated pipetting in a PCR tube. The amplification procedure was performed according to the following thermal profile: initial denaturation at 95°C for 10 min, followed by 40 cycles of 95°C for 10 min, 60°C for 30 s, 72°C for 30 s and a final extension step at 72°C for 5 min. Amplified products were separated on 1.5% agarose gel stained with SafeDye (ADD BIO INC, Daejeon, Republic of Korea, Cat. No. A4671) and visualized under UV illumination. A 100 bp DNA ladder was used as a molecular weight marker (Figure 2).

### 2.7 Statistical Analysis

Descriptive statistical analyses were conducted using Microsoft Excel Office 2010. Chi‐square tests were employed to evaluate the significance of differences in prevalence across various categorical factors, including farm location, age group (≤1 year, 1–3 years, 3–5 years and >5 years), sex (male and female) and health status (apparently healthy vs. diarrhoeic). A *p* value of less than 0.05 was considered statistically significant. All inferential statistical analyses were performed using SPSS (SPSS Inc., Chicago, IL, USA).

The primary outcome variables—bTB status (positive or negative)—were determined using both serological (ELISA) and molecular (PCR) diagnostic methods. For ELISA, animals were classified as positive or negative based on the manufacturer's recommended cutoff values: Absorbance values above the cutoff were considered positive, whereas those below were classified as negative. PCR results were interpreted similarly, with animals deemed positive if specific target DNA amplification was detected and negative if no amplification occurred.

Both ELISA and PCR outcomes were coded as dichotomous variables (positive = 1, negative = 0). Descriptive statistics, including frequencies and proportions, were used to summarize the data. Logistic regression analysis was applied to identify significant predictors of ELISA and PCR positivity, adjusting for potential confounding variables as described by Hoque et al. (2023). Furthermore, binomial exact methods were used to calculate 95% CIs for estimated proportions of bTB positivity.

## 3 Results

### 3.1 Molecular and Serological Prevalence of *Mycobacterium bovis*

The prevalence of *M. bovis* was determined through both molecular and serological methods. Of the 500 samples tested, the overall prevalence of *M. bovis* was 10.6%. Among the 250 serum samples tested, 8.0% (20/250, 95% CI: 4.95–12.08) were positive by ELISA, whereas the PCR testing on milk samples showed a higher prevalence of 14.8% (37/250, 95% CI: 10.6–19.8). Additionally, PCR testing on blood serum samples detected 6.4% (16/250, 95% CI: 3.7–10.2) of animals as positive for *M. bovis* (Table 1).

**TABLE 1:** Both molecular and sero‐prevalence of *Mycobacterium bovis* in cattle at Sylhet Division of Bangladesh.

Test method	Samples	*x*/*N*	Prevalence (%)	95% CI (lower bound, upper bound)
Indirect ELISA	Blood	20/250	8.0	4.95, 12.08
PCR	Milk	37/250	14.8	10.6, 19.8
	Blood	16/250	6.4	3.7, 10.2
	Overall	53/500	10.6	8.04, 13.6

*Note: N*, number of samples tested; *x*, number of positive samples.

Abbreviation: CI: confidence interval.

### 3.2 Prevalence and Associated Risk Factors

#### 3.2.1 Prevalence and Farm‐Associated Risk Factors

Significant variation in sero‐prevalence of *M. bovis* was observed across different farm locations in Sylhet and Sunamganj districts (Table 2). In Sylhet district, farm‐level sero‐prevalence ranged from 0% (F3 and F6) to 10.0% (F4 and F5), with an overall prevalence of 5.3% (8/150, 95% CI: 2.3–10.2). In contrast, cattle sampled from individual farms in Sunamganj district exhibited a higher prevalence of 12.0% (12/100, 95% CI: 6.4–20.0). The overall sero‐prevalence across all farms in the study area was 8.0% (20/250, 95% CI: 4.9–12.1). A statistically significant difference in prevalence was noted among farms in Sylhet district (*p* < 0.05), suggesting that geographic and management‐related farm factors may contribute to the distribution of infection.

**TABLE 2:** Sero‐prevalence of *Mycobacterium bovis* in association with different farm's location.

Farm location	Specific farm identification	*x*/*N*	Prevalence (%)	95% CI (lower bound, upper bound)	*p* value
Sylhet district	Farm‐1 (F1)	3/40	7.5	1.6, 20.4	<0.01
	Farm‐2 (F2)	1/20	5.0	0.1, 24.9	
	Farm‐3 (F3)	0/20	0	N/A	
	Farm‐4 (F4)	2/20	10.0	1.2, 31.7	
	Farm‐5 (F5)	1/10	10.0	0.3, 44.5	
	Farm‐6 (F6)	0/20	0	N/A	
	Farm‐7 (F7)	1/20	5.0	0.1, 24.9	
	Total	8/150	5.3	2.3, 10.2	
Sunamganj district	Cattle from individual farms	12/100	12.0	6.4, 20.0	
Total	250	20/250	8.0	4.9, 12.1	

*Note*: Level of significance *p *< 0.05; *N*, number of samples tested; *x*, number of positive samples.

Abbreviation: CI: confidence interval.

#### 3.2.2 Prevalence and Host‐Associated Risk Factors

##### 3.2.2.1 Age

Age significantly influenced the infection status of *M. bovis* (*p *= 0.02). Using calves ≤1 year as reference group (prevalence: 3.65%), the 1–3 years age group showed the higher prevalence at 17.51% (24/137; 95% CI: 1.06–9.5) with an odds ratio (OR) of 3.16 (95% CI: 1.06–9.5). However, no significant differences were observed for the 3–5 years (7.52%, 7/93) and >5 years (10.10%, 19/188) age groups, with ORs of 0.52 (95% CI: 0.27–1.02) and 0.97 (95% CI: 0.52–1.84), respectively (Table 3).

**TABLE 3:** Prevalence of *Mycobacterium bovis* in association with age, sex, breed and health status of animals.

Age of animals	*x*/*N*	Prevalence (%)	Coefficient	*p* value	Odds ratio (OR)	95% CI
Constant			2.27	<0.001		
Age						
Up to 1 year	3/82	3.65	Ref.			
1–3 years	24/137	17.51	1.15	0.04	3.16	1.06–9.5
3–5 years	7/93	7.52	−0.66	0.22	0.52	0.27–1.02
>5 years	19/188	10.10	−0.024	0.92	0.97	0.52–1.84
Sex of animals						
Male	14/213	6.57	Ref.			
Female	39/287	13.58	−0.87	<0.001	0.42	0.23–0.78
Breed						
Indigenous	12/180	6.67	Ref.			
Crossbred	34/270	12.59	−0.23	0.19	0.79	0.53–1.20
Holstein Friesian	7/50	14.0	−0.75	<0.001	0.47	0.26–0.84
Health status						
Apparently healthy	22/341	6.45	Ref.			
Diarrhoeic	31/159	19.49	1.41	<0.001	4.09	2.27–7.39

*Note*: Level of significance *p* < 0.05; *N*, number of samples tested; Ref.: reference category; *x*, number of positive samples.

Abbreviation: CI: confidence interval.

##### 3.2.2.2 Sex

Female cattle had a significantly higher prevalence (13.58%, 39/287) than males (6.57%, 14/213), with males exhibiting a lower likelihood of infection (OR = 0.42; 95% CI: 0.23–0.78; *p* < 0.001) (Table 3).

##### 3.2.2.3 Breed

Breed type showed a significant relationship with infection prevalence. Holstein Friesian cattle had the highest prevalence (14.0%, OR = 0.47, 95% CI: 0.26–0.84), followed by crossbred cattle (12.59%, OR = 0.79, 95% CI: 0.53–1.20), indicating a significantly lower likelihood of testing positive compared to indigenous cows (Table 3).

##### 3.2.2.4 Health Status

Health condition was strongly associated with *M. bovis* positivity. Diarrhoeic animals exhibited a much higher prevalence of 19.49% (31/159), with an OR of 4.09 (95% CI: 2.27–7.39; *p *< 0.001), indicating that diarrhoeic cows were more likely to test positive for *M. bovis* than apparently healthy animals, which had a prevalence of 6.45% (22/341) (Table 3).

## 4 Discussion

In the present study, 250 blood and 250 milk samples were collected from Sylhet and Sunamganj districts of Sylhet Division to assess the prevalence of *M. bovis* using both serological (ELISA) and molecular (PCR) methods. ELISA is a practical tool for large‐scale screening due to its cost‐effectiveness and ease of use; however, it detects antibodies rather than the pathogen, making it less reliable for early or latent infections. False positives may occur from cross‐reactivity or prior vaccination, and antibody levels can vary with host response. PCR on milk allows for direct detection of *M. bovis* and is more sensitive during active shedding. However, its accuracy can be compromised by PCR inhibitors in milk, low bacterial loads and inconsistent shedding, making results highly dependent on sample quality and processing efficiency (Al‐Farha et al. 2020; Collins 2015).

In this study, the serological testing revealed that 20 out of 250 blood samples were positive for *M. bovis* with an apparent prevalence of 8.0%. This rate is moderately higher than findings reported in Chattogram (5.9%) and Mymensingh (5.9%) (Islam et al. 2021; Mondal et al. 2014). The slightly elevated prevalence in Sylhet may be attributed to regional differences in farm management practices, animal movement or biosecurity measures. In regions with less stringent practices, higher prevalence rates are often observed (Islam et al. 2021). Conversely, our finding is comparable to the 7.8% prevalence observed in Sirajganj (Mahmud et al. 2014), indicating potential similarities in herd composition, disease control efforts or diagnostic sensitivity.

PCR testing of milk samples in this study showed a higher prevalence of *M. bovis* (14.8%), aligning closely with the 14% reported by Mdegela et al. (2004). However, this rate is somewhat lower than the 19% detected by Durnez et al. (2009) but higher than the findings of Islam et al. (2021). These differences could stem from variations in sampling techniques, stage of infection, pathogen load in milk or the presence of PCR inhibitors in dairy matrices (Tawab et al. 2019; Zumárraga et al. 2012). Previous studies have shown that *M. bovis* DNA detection in milk can vary widely (2%–87%) depending on region, herd health and test sensitivity (Borges et al. 2016).

In serological testing, out of the 250 milk samples collected from two different districts, F4 and F5 in Sylhet district exhibited the highest sero‐prevalence of 10%, whereas individual farmers in Sunamganj district showed a prevalence of 12% in their cattle. The results indicated that the prevalence rate of *M. bovis* was statistically significant (*p* = <0.01).

This study also identified several host‐associated risk factors for bTB. Age was significantly associated with infection status. The prevalence of *M. bovis* was higher among cattle aged 1–3 years, estimated to be 17.51%. These age‐related findings differ from those reported by other researchers (Cleaveland et al. 2007; Tschopp et al. 2009). The elevated susceptibility in younger adults may reflect their immunological naivety or recent exposure during herd expansion or movement (Augusta et al. 2024; Gupta et al. 2024). Health status also influenced bTB prevalence. Diarrhoea was considered a health indicator due to its visibility and relevance to systemic infections. In this study, the diarrhoeic animals with respiratory symptoms have shown higher percentage (19.49%) of prevalence than the healthy animals, which is in contrast with the previous findings (Katale et al. 2013; Munyeme et al. 2009). This discrepancy may result from differences in case definitions, diagnostic criteria or herd management (Chaudhari et al. 2024; Sarangi et al. 2023; Xu et al. 2021).

Sex was another significant factor; females exhibited a higher prevalence (13.58%) compared to males (6.57%), which agrees with findings by Inangolet et al. (2008). This difference may be due to prolonged retention of females in dairy herds for reproduction and milk production, increasing their cumulative exposure to infection sources over time (Trangadia et al. 2013).

The study highlights a notable difference in sero‐prevalence between Sylhet and Sunamganj districts, with Sunamganj showing a higher prevalence. This suggests that geographic factors, possibly including environmental conditions and local farming practices, may influence the spread of *M. bovis* (Nath et al. 2023; Islam et al. 2020).

PCR amplification targeting the IS6110 gene consistently produced a 150 bp fragment in positive cases, with no amplification in non‐mycobacterial controls. This indicates high specificity of the assay for members of the *M. tuberculosis* complex.

This study highlights the significant impact of age, sex, breed and health status on the prevalence of *M. bovis* in cattle in the Sylhet Division of Bangladesh. Specifically, age groups ‘1–3 years’ and ‘3–5 years’, along with female sex, Holstein Friesian breed and diarrhoeic health status, were associated with an increased risk of *M. bovis* positivity.

Our findings provide evidence of the prevalence of *M. bovis* DNA in cow samples. This study shows a significant rate of bTB in some dairy‐intensive and extensive areas of Sylhet and Sunamganj. It indicates that conducting active surveillance and developing a national strategy for bTB elimination is important to reduce disease transmission between animals and to humans. Diarrhoeic cows with respiratory distress could be targeted in prospective bTB surveillance programs.

In conclusion, our findings demonstrate the endemic presence of *M. bovis* in dairy herds of Sylhet Division, with significant associations with age, sex, breed and health status. These results underscore the need for ongoing active surveillance, particularly in high‐risk herds and the implementation of a national bTB control strategy.

Although the findings are interesting, this study was limited by its cross‐sectional design, which prevents assessment of temporal trends or causality. Future studies should include body condition score and other clinical indicators, including diarrhoea. Given the zoonotic potential of bTB, targeted interventions are essential to safeguard both animal and public health.

## Author Contributions

**Md. Atik Faysal**: data collection, sample collection and processing, conducting experiment, formal analysis, methodology, writing – original draft, writing review and editing. **Md. Shahidur Rahman Chowdhury**: formal analysis, writing – original draft, writing review and editing. **Fatema Yeasmin Tanni**: formal analysis, writing – original draft, writing review and editing. **Hemayet Hossain**: formal analysis, software, writing – original draft, writing review and editing. **Khadiza Akter Brishty**: formal analysis, writing – original draft, writing review and editing. **Md. Bashir Uddin**: conceptualization, methodology, investigation, writing – original draft, supervision, writing review and editing. **Md. Masudur Rahman**: methodology, investigation, writing – original draft, writing review and editing. **Md. Mahfujur Rahman**: investigation, methodology, writing – original draft, supervision, writing review and editing. **Md. Mukter Hossain**: conceptualization, methodology, investigation, supervision, writing – original draft, formal analysis, writing review and editing. All authors have read and approved the final version of the manuscript.

## Ethics Statement

Blood and milk samples used in this study were obtained by oral consent of the local authorities of intensive commercial dairy farm or from owners of the free‐grazing animals found in the field, in complete agreement with Bangladesh animal act legislation. No animals were put to death especially for this investigation. No ethical approval was thought to be required.

## Conflicts of Interest

The authors declare no conflicts of interest.

## Peer Review

The peer review history for this article is available at https://www.webofscience.com/api/gateway/wos/peer‐review/10.1002/vms3.70531.

## Data Availability

All data generated and analysed in this study are included in the main manuscript.
